# CO_2_ Laser Therapy for Genitourinary Syndrome of Menopause in Women with Breast Cancer: A Randomized, Sham-Controlled Trial

**DOI:** 10.3390/cancers17071241

**Published:** 2025-04-06

**Authors:** Sireen Jaber, Gabriel Levin, Maya Ram-Weiner, Ahinoam Lev-Sagie

**Affiliations:** 1 Department of Obstetrics and Gynecology, Hadassah-Hebrew University Medical Center, Jerusalem 9765422, Israel; sireen.jaber@health.nsw.gov.au (S.J.); mawei@hadassah.org.il (M.R.-W.); 2 The Department of Gynecologic Oncology, Hadassah Medical Center, Faculty of Medicine, Hebrew University of Jerusalem, Jerusalem 9190500, Israel; gabriel.levin2@mail.mcgill.ca; 3 Lady Davis Institute for Cancer Research, Jewish General Hospital, McGill University, Montreal, QC H3A 0G4, Canada; 4 Clalit Health Organization, Jerusalem 9780214, Israel; 5 Faculty of Medicine, Hebrew University of Jerusalem, Jerusalem 9190500, Israel

**Keywords:** vulvovaginal atrophy, genitourinary syndrome of menopause, breast cancer, dyspareunia, fractional CO_2_ laser

## Abstract

Objectives: We aimed to study the efficacy of fractional CO_2_ laser for genitourinary syndrome of menopause (GSM) symptoms in breast cancer (BC) survivors through a randomized, sham-controlled study, followed by an open-phase study assessing the impact of additional treatments. Methods: BC survivors with GSM were randomized to receive either three sessions of intravaginal CO_2_ laser or sham treatment every 3–4 weeks. The laser’s energy was 45–60 mJ/pixel. Outcomes were compared one month following the last session. Participants initially receiving laser treatment were offered three more sessions, while those receiving sham had six laser sessions in an open-label study. Results: Thirty-four BC survivors were randomized to laser (n = 19) or sham (n = 15) treatments. Dyspareunia and intercourse dryness scores improved in both groups one month post-treatment, without a significant advantage of laser over sham. The laser treatment resulted in a reduction in daily dryness (−1.30 ± 0.55, *p* = 0.017), an increase in vaginal hydration (3.24 ± 1.13, *p* = 0.004), and an increase in Vaginal Health Index (VHI) (2.26 ± 0.50, *p* < 0.001). Most participants (18/19 and 9/15, respectively) opted to continue laser treatments after unblinding, resulting in 27 patients receiving six laser treatments. Increasing the number of laser treatments was associated with a constant improvement in Visual Analogue Score (VAS) scores for dyspareunia, intercourse dryness, daily dryness, burning, discomfort, itch, and average VAS, as well as pH, VHI, and hydration. Conclusions Three fractional CO_2_ laser treatments for BC survivors reduced daily dryness but did not improve dyspareunia and sexual dryness when compared to sham in this randomized trial. Increasing the number of treatment sessions seemed to improve outcomes; however, it remained clinically insufficient, even after six treatments.

## Simple Summary

Genitourinary syndrome of menopause, associated with dyspareunia and daily genital inconvenience, negatively affects breast cancer survivors’ quality of life. Fractional CO_2_ laser therapy is increasingly used to treat this common condition, albeit conclusive evidence of its effectiveness is still lacking. We conducted a randomized, sham-controlled trial of three laser treatments, followed by an open-phase study, evaluating up to six total treatments. The initial three treatments showed no significant difference from sham treatment. While increasing the number of treatment sessions to six showed modest improvement, outcomes remained below the threshold for clinical significance. Further evaluation of optimal treatment protocols, patient selection criteria, and long-term outcomes is needed before integrating fractional CO_2_ laser therapy into routine care for breast cancer survivors with GSM.

## 1. Introduction

Advancements in the diagnosis and treatment of breast cancer (BC) have led to a significant decrease in its mortality [1]. Adjuvant anti-estrogenic therapy (aromatase inhibitors (AIs), tamoxifen, and GnRH-agonists) is frequently used to produce an estrogen-blockade state and reduce recurrence [2]. Consequently, more BC survivors suffer from estrogen deficiency sequelae and menopausal symptoms. Up to 57% of BC survivors suffer from genitourinary syndrome of menopause (GSM) [2], secondary to estrogen deficiency in the urogenital epithelium, involving the vulva, vagina, and lower urinary tract [3]. Symptoms include vulvovaginal dryness, discharge, irritation, burning, disturbances of sexual function, including impaired lubrication and dyspareunia, and urinary urgency, frequency, dysuria, and recurrent infections [3].

GSM symptoms tend to be more severe, more abrupt in onset, and more persistent in BC survivors [2,4], negatively affecting patients’ quality of life [3]. While long-term survivors often report the normalization of physical and emotional functioning, they experience continued difficulty with sexual functioning and satisfaction for five years or more after treatment [5,6], with an estimated 20% of patients consider terminating or terminating adjuvant endocrine therapy [7].

Topical estrogen therapy for GSM is controversial in BC survivors due to potential systemic absorption [6,8]. Consequently, many BC patients and physicians are reluctant to use these preparations. Non-hormonal vaginal moisturizers and lubricants often prove unsatisfactory, especially with severe symptoms [6,7].

In recent years, energy-based devices have been suggested as treatments for GSM [9]. One such modality, fractional CO_2_ laser therapy, is proposed to initiate neovascularization [10], collagen and extracellular matrix production, and growth factor tissue infiltration, resulting in the improvement of epithelial thickness and the restoration of elasticity and moisture [11]. Previously, the energy levels used for pixel CO_2_ lasers ranged between 40 and 120 mJ/pixel [10,12]. It was suggested to adjust energy levels according to the vaginal wall thickness, which varies with menopause duration [13].

Energy-based treatments are an attractive option that avoid hormonal interventions, a potential advantage for BC survivors. Observational trials have demonstrated that intravaginal laser-based devices effectively relieve GSM symptoms in BC survivors [14,15,16,17,18,19,20,21,22,23,24]. Sham-controlled, randomized, blinded trials are scarce and often inconclusive [25,26,27,28,29]. Given the need for an effective non-hormonal treatment for GSM in BC survivors, we conducted a prospective, randomized, single-blind, controlled trial to assess the efficacy of CO_2_ laser therapy for treating GSM in BC survivors.

## 2. Materials and Methods

The study included two phases: first, we compared three CO_2_ laser treatments to three sham sessions; second, we assessed outcomes of six laser treatments in an open-phase study. The study was approved by the Hadassah Medical Center Institutional Review Board (approval number: HMO-0392-18). Written informed consent was obtained from all subjects.

Participants were recruited from BC oncology clinics and underwent eligibility screening at a specialized clinic for vulvovaginal disorders. BC survivors with bothersome GSM symptoms (see below), reporting intercourse dryness and/or dyspareunia ≥5 in severity using a Visual Analogue Scale (VAS) were included. Additional inclusion criteria were a diagnosis of BC, age > 18, menopause, microscopic confirmation of vaginal atrophy, and a normal cervical screening test within the last 3 years. Vaginal atrophy was defined as thin, dry, vaginal epithelium, pH measurement >5, and microscopic smear showing abundant parabasal cells. Exclusion criteria included vaginal bleeding, current chemotherapy or radiation therapy, treatment with systemic or vaginal estrogen within the year prior to enrollment, and previous diagnosis of genital dysplasia. All participants completed a questionnaire providing details on demographics, medical history, gynecological history, GSM symptoms, and previous GSM treatment (systemic, topical hormonal, and non-hormonal treatment). Patients who had been regularly using vaginal lubricants and moisturizers before enrollment were allowed to continue using them if they reported persistent symptoms despite regular use.

### 2.1. Study Protocol (Figure 1)

During the screening visit (T0), patients were randomly assigned to receive either CO_2_ laser treatment or a sham treatment in a 1:1 ratio using a computer algorithm. A gynecological examination was performed, and study parameters were evaluated (see below).

**Figure 1:**
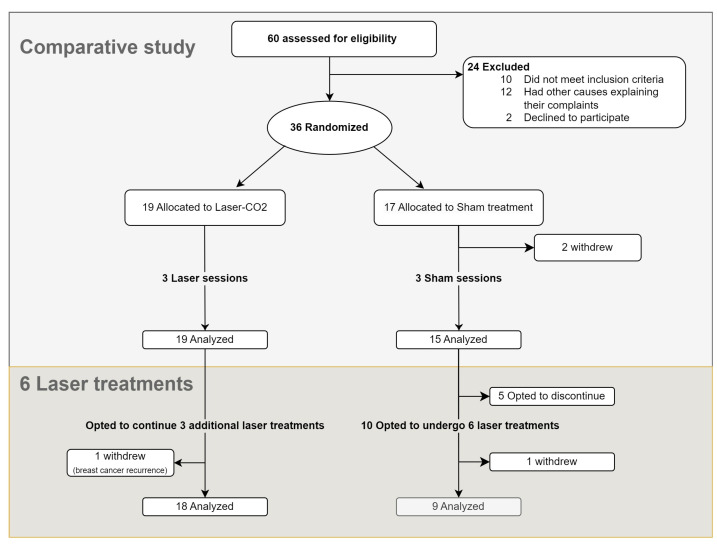
Study’s flowchart.

Participants were blinded to their treatment allocation. The treatment group underwent three laser treatments every 20–30 days (T1–T3). The sham group underwent a similar procedure with the same equipment, without active laser energy. A single practitioner (the last author, ALS), unblinded to the therapy, performed the laser/sham treatments. Following three laser/sham treatments, participation in an open-phase study was offered to the patients; those who initially received laser and were not satisfied with treatment results were offered three additional treatments (T3–6), while those who initially received sham and were interested were offered six laser treatments conducted every 20–30 days (Figure 1).

During visits, participants completed VAS questionnaires and underwent a gynecological examination to assess vaginal pH, Vaginal Health Index (VHI) score, and vaginal hydration (see below). Four weeks after the third laser/sham treatment, a follow-up visit was conducted, and clinical evaluation was performed (these data are defined “end of phase 1”, EOP1).

In those who participated in the open-phase study, follow-up evaluations were conducted at 1 (termed FU1), 3, and 6 months following the final treatment. Data from the 3rd and 6th months follow-up visits are not shown, given that no clinically significant change was found one month after treatment completion.

### 2.2. Treatment with CO_2_ Laser and Sham

The procedure was carried out in the lithotomy position in an identical room, with the same set-up, by a single experienced practitioner. Prior to the treatment, 5 mL of 10% lidocaine in Vaseline base was applied to the vagina and vestibule for 30 min in both groups to minimize discomfort. The use of topical anesthesia prevented pain or heat sensation during treatment and contributed to the participants’ blinding.

The treatment was conducted using the FemiLift™ fractional microablative CO_2_ laser system (Alma Lasers, Israel), which delivers CO_2_ laser energy to the vaginal tissue via a diffractive optical element that pixelates the beam to 81 microscopic pixels in a 9 × 9 pattern. The specialized probe was inserted into the vagina and treatment was carried out according to the usual protocol, using pulse mode. Although we aimed at energy levels of 45–80 mJ/pixel, the actual range was 45–60 mJ/pixel, as most women could not tolerate the pain associated with higher energy levels even with the usage of topical anesthesia. The probe was rotated in a 360-degree pattern to cover the entire circumference and length of the vagina, including the vestibule. The treatment was repeated three times, from the apex of the vagina to the introitus. The suppression of auditory and visual effects was performed in both groups to ensure participant blinding. A designated aspirator was used during the procedure to mask olfactory stimulus from vaporization and smoke production.

### 2.3. Outcome Measures Included

- Vaginal pH: using an indicator strip (range 4–8).
- VHI score (Table S1): Evaluating five parameters (elasticity, secretions, pH, epithelial integrity, hydration) aimed to define vaginal atrophy degree [30]. Total score ranges from 5 to 25, with atrophy defined as <15.
- Vaginal hydration measured by the modified Schirmer test [31]: Measurement was performed by placing a calibrated filter paper strip (Schirmer tear test strips) in the vaginal introitus in a standard manner [31] for 5 min. The paper was removed, and the amount of moisture was measured in millimeters. This measure allows for the direct and objective evaluation of vaginal hydration.
- VAS assessments for GSM symptoms: Dryness during intercourse (“intercourse-dryness”), dyspareunia, daily vaginal dryness, discomfort, burning, discharge, itching, and dysuria. The severity of the symptoms was measured using an eleven-point VAS, with 0 representing “absence of symptom”, and 10 being “most severe symptom”.
- Female Sexual Function Index (FSFI): The FSFI is a nineteen-item questionnaire that assesses six domains of sexual function in women: desire, arousal, lubrication, orgasm, satisfaction, and pain. Each item is scored on a scale of 0 or 1 to 5, with higher scores indicating better sexual function. The total FSFI score ranges from 2 to 36, with a score of 26.55 or less suggesting sexual dysfunction. Both the individual domain scores and the total scores were analyzed, to provide a view of sexual function across different areas as well as the overall assessment [32]. The FSFI has been validated in BC patients [33].

The co-primary outcomes included the change in severity of intercourse dryness and dyspareunia as measured by the VAS score. Secondary outcome measures included change in severity of all other GSM symptoms, as well as changes in the FSFI scores, pH levels, VHI scores, and vaginal hydration.

Comparisons were conducted for Phase 1 (laser vs. sham), comparing measures at baseline and at one-month follow-up after three sessions, and for Phase 2 (six laser treatments), comparing the changes over time in relation to baseline.

### 2.4. Statistical Analysis

The data analysis involved collecting detailed subject-specific information across multiple visits to assess the effects of laser intervention compared to sham treatment. In Phase 1, we analyzed laser versus sham effects at multiple time points (T1, T2, T3, and EOP1). In Phase 2, participants were analyzed as a single cohort with measurements at FU1 compared to baseline.

Normality Testing: We performed normality testing for all scales at both baseline and at EOP1 using the Shapiro–Wilk test, chosen for its sensitivity in detecting departures from normality in small-to-moderate sample sizes.

Descriptive and Inferential Statistics: Continuous variables were summarized using mean ± SD, and comparisons between laser and sham treatments were carried out using the Mann–Whitney U test given the non-normal distribution of data. Categorical variables were summarized using counts and percentages, with comparisons between groups conducted using Fisher’s exact test.

Generalized Estimating Equations (GEE): In Phase 1, we utilized the GEE model to assess the influence of both treatment and time on outcomes, while accommodating the correlation of repeated measurements within subjects. The GEE model is robust against deviations from normality in continuous data and is particularly suited for longitudinal data. This approach allows for the analysis of repeated measurements over time, effectively handling the within-subject correlation. The initial model included both time as a categorical variable representing the visits and an interaction term for time and treatment group. However, since all interactions were found to be non-significant, we simplified the approach to use only the main effects. We used an identity link function and a Gaussian family with an exchangeable correlation structure for this analysis.

Phase 2 Analysis: For Phase 2, involving six laser treatments, only the time effect was evaluated, as all participants received treatment. We continued to use the GEE model framework to analyze these effects, emphasizing the time variable’s impact over the course of the treatments.

Statistical significance was determined by two-sided *p*-values less than 0.05.

The sample size was calculated (SPSS, V 29.02) according to previous data [17], showing that the most bothering symptoms are dryness and dyspareunia, and using the following inputs: effect size (Cohen’s D): 0.8, based on a meaningful difference in mean VAS scores from 9 (before treatment) to 5 (after treatment); standard deviation (SD): 5; delta (mean difference): 4; (α): 0.05; power: 80%. The calculated sample size per group was 26. To account for potential dropout, we planned to recruit 30 participants per group.

In 2020–2022, the COVID-19 pandemic severely affected trial recruitment and conduct and forced early trial cessation before the accrual of the initially planned number of participants could be completed, despite the efforts of the investigators. Given a minimal dropout (2/36 patients), a suspension of further recruitment was decided.

The analyses were carried out using R-4.4.1 (R Foundation for Statistical Computing, Vienna, Austria).

## 3. Results

Between April 2019 and June 2022, 60 patients were screened, of which 36 were recruited (Figure 1). Two patients in the sham group withdrew before the completion of three sessions. Ultimately, 34 patients completed the comparative study, of whom 19 patients were assigned to the laser group and 15 to the sham group.

### 3.1. Baseline Characteristics

Patients’ demographics are presented in Table 1, and BC treatments are presented in Table S2.

The two groups did not differ in terms of age, BMI, medical history, BC treatments, menopause cause, or duration. Most participants (n = 26, 76%) had received adjuvant anti-estrogenic therapy, mostly AI (n = 21, 61%). Eight women (23.5%) previously used hormonal treatment (Table S3).

At baseline, the two groups did not differ regarding the various measures, except for dryness, which was worse in the sham group (Table S4). The scores for dyspareunia and intercourse dryness were higher than those of the remaining GSM symptoms (Figure S1).

### 3.2. Comparison of CO_2_ Laser to Sham (Phase 1, Three Sessions)

#### 3.2.1. Primary Outcomes (Figure 2)

Both dyspareunia and intercourse dryness improved over time in both groups, with no significant advantage for the laser over sham.

**Figure 2:**
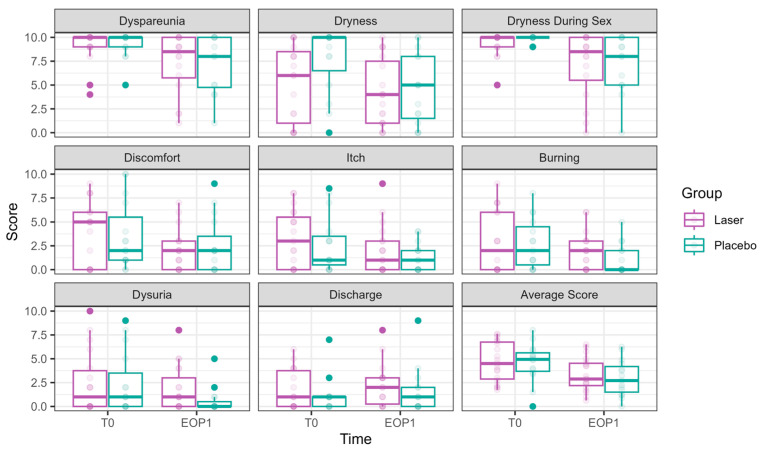
VAS scores at Phase 1 (comparative study). VAS scores at T0 are presented in the left bar (purple—laser, green—sham), and those at EOP1 are presented in the right bar. (VAS—visual analog scale, T0—before intervention, EOP1—end of phase 1).

The following is a report of these outcomes, structured by time effect and laser effect.

Dyspareunia significantly improved over time. By T3, the reduction was −1.56 ± 0.64, *p* = 0.015, with further improvement at EOP1 (−1.89 ± 0.64, *p* = 0.003). Nevertheless, no significant effect of laser treatment was observed (0.12 ± 0.43, *p* = 0.777).

Vaginal dryness during sexual intercourse significantly decreased at T2 (−1.66 ± 0.53, *p* = 0.002), T3 (−2.34 ± 0.57, *p* < 0.001), and EOP1 (−2.38 ± 0.62, *p* = 0.0001). However, no significant effect of laser treatment was observed (−0.03 ± 0.40, *p* = 0.944).

#### 3.2.2. Secondary Outcomes (Figure 2, Figure 3 and Figure 4 and Tables S4–S6)

The following is a report of secondary outcomes, structured by time effect and laser effect.

Daily dryness significantly improved by EOP1 (−2.00 ± 0.88, *p* = 0.022). Laser treatment had a significant effect on dryness reduction (−1.30 ± 0.55, *p* = 0.017).

Itch significantly improved by EOP1 (−1.34 ± 0.61, *p* = 0.027). Nevertheless, laser treatment had the opposite effect, significantly increasing itch (0.84 ± 0.39, *p* = 0.033)

Discomfort significantly improved at EOP1 (−1.41 ± 0.70, *p* = 0.044), without an additional laser effect (0.09 ± 0.48, *p* = 0.857).

Dysuria showed a borderline improvement by EOP1 (−1.15 ± 0.64, *p* = 0.073), with an opposite effect for laser treatment, showing significantly higher values compared to sham (1.26 ± 0.42, *p* = 0.003).

Discharge showed no improvement over time (0.45 ± 0.51, *p* = 0.375). The laser had the opposite effect, with discharge symptoms significantly higher compared to sham (1.14 ± 0.36 *p* = 0.002).

Burning was significantly reduced by EOP1 (−1.30 ± 0.59, *p* = 0.027). Laser treatment had an opposite effect (0.96 ± 0.37, *p* = 0.009).

The average VAS score decreased significantly over time, with an improvement at EOP1 (−1.44 ± 0.46, *p* = 0.0015). No significant additional effect of laser treatment was found (0.42 ± 0.30, *p* = 0.165).

**Figure 3:**
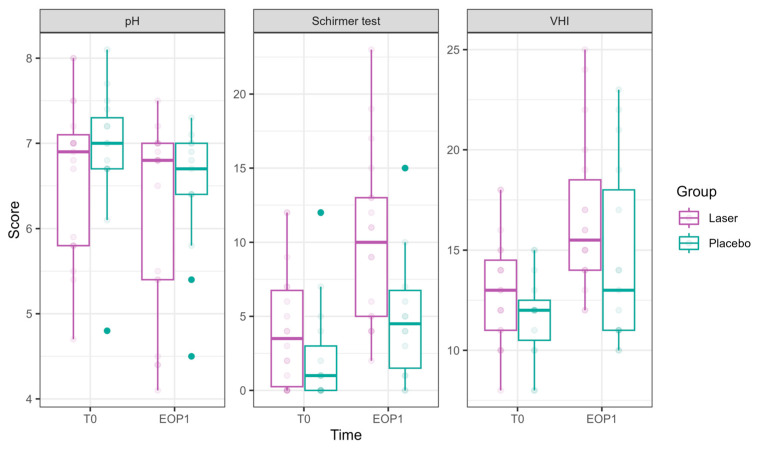
pH, hydration, and VHI parameters at Phase 1 (comparative study). Parameters of pH, hydration (as measured in millimeters by the Schirmer modified test), and VHI (Vaginal Health Index) at T0 are presented in the left bar (purple—laser, green—sham), and those at EOP1 are presented in the right bar. (VHI—Vaginal Health Index, T0—before intervention, EOP1—end of phase 1).

pH showed a significant reduction at EOP1 (−0.46 ± 0.22, *p* = 0.041), accompanied by a significant laser effect of pH improvement compared to sham (−0.31 ± 0.14, *p* = 0.021).

VHI showed significant improvements at T2 (1.53 ± 0.73, *p* = 0.036), T3 (2.71 ± 0.75, *p* = 0.0003), and EOP1 (3.35 ± 0.83, *p* < 0.001). In addition, laser treatment significantly improved VHI (2.26 ± 0.50, *p* < 0.001).

Schirmer test scores representing hydration significantly improved at EOP1 (4.73 ± 1.15, *p* < 0.001). Additionally, laser treatment significantly improved its results compared to sham (3.24 ± 1.13, *p* = 0.004).

**Figure 4:**
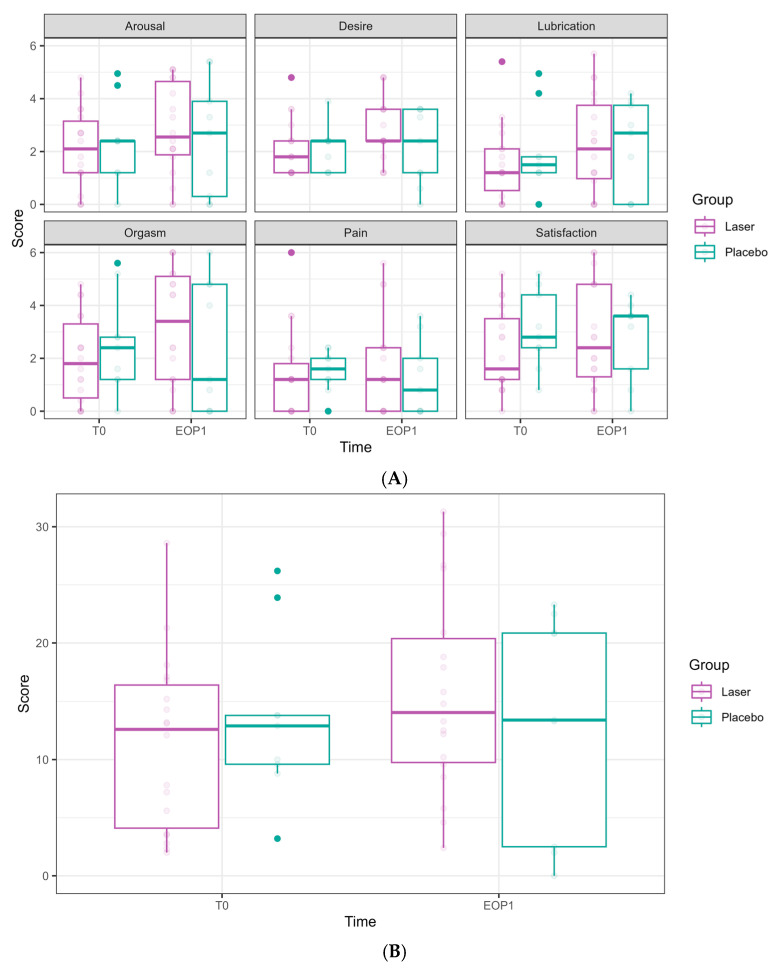
Female Sexual Function Index (FSFI) domain scores at Phase 1 (comparative study). (**A**) FSFI domains are presented separately. Scores at T0 are presented in the left bar (purple—laser, green—sham), and those at EOP1 are presented in the right bar. (T0—before intervention, EOP1—end of phase 1). (**B**) Total score of FSFI. Score at T0 is presented in the left bar (purple—laser, green—sham), and score at EOP1 is presented in the right bar (T0—before intervention, EOP1—end of phase 1).

The FSFI scores (relating to arousal, desire, lubrication, orgasm, pain, satisfaction, and the overall FSFI summarized score) did not show any significant effects from either time or laser treatment (Figure 4).

### 3.3. Outcomes of Six CO_2_ Laser Treatments (Phase 2)

**Figure 5:**
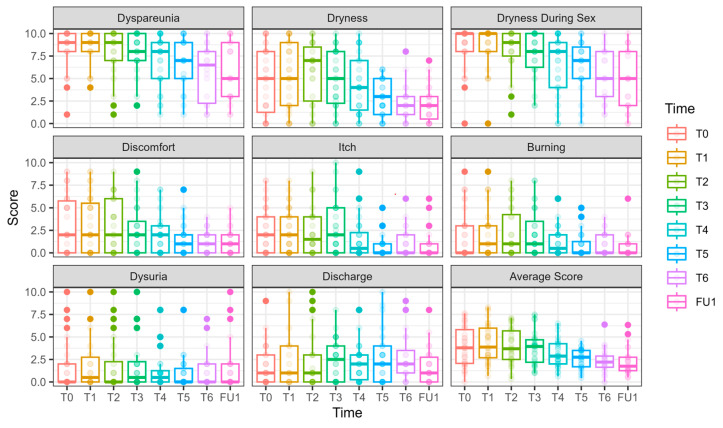
VAS scores along the longitudinal study of 6 laser treatments, T0-FU1. VAS scores are presented for 27 women who underwent 6 laser sessions (T0, initial assessment, FU1—one month after completion of 6 laser treatments, designated T1–T6). Although there is a continuous and gradual reduction in dyspareunia and dryness during intercourse, the scores remain high.

**Figure 6:**
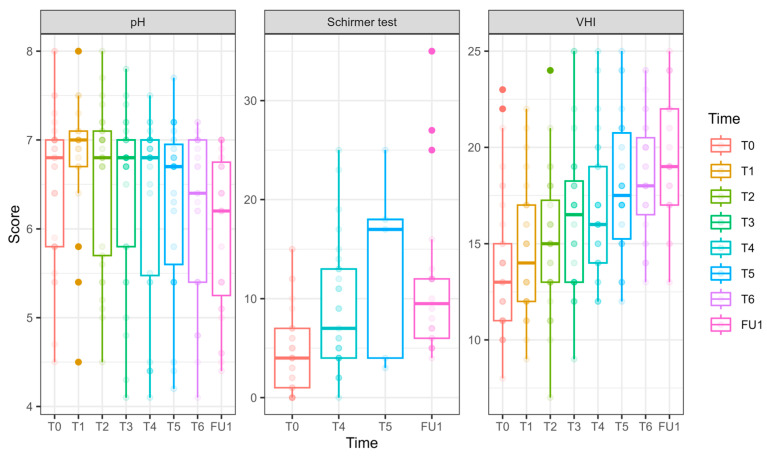
pH, hydration, and VHI parameters along the longitudinal study of 6 laser treatments, T0 to FU1. Parameters of pH, hydration (as measured in millimeters by the Schirmer modified test), and VHI (Vaginal Health Index) are presented for 27 women who underwent 6 laser sessions (T0, initial assessment, FU1—one month after completion of 6 laser treatments, designated T1–T6). The reduction in pH is clinically insignificant (and remains high); the hydration as measured by the modified Schirmer test has gradually increased over the treatments; the Vaginal Health Index (VHI) score has increased throughout the treatments.

**Figure 7:**
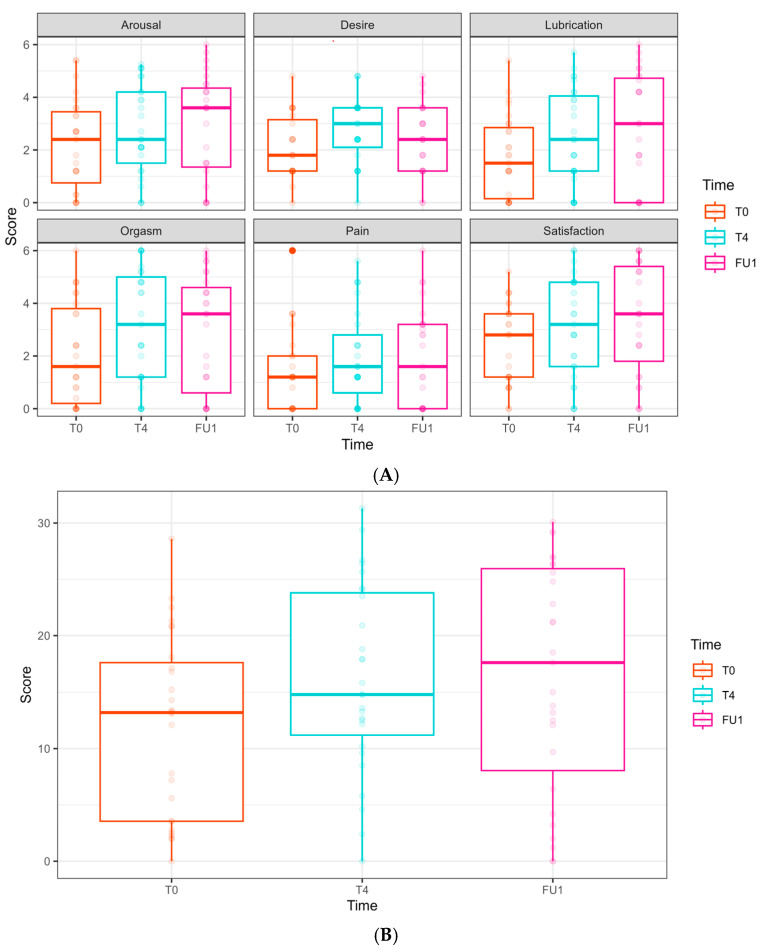
Female Sexual Function Index (FSFI) domain scores along the longitudinal study of 6 laser treatments, T0 to FU1. (**A**) FSFI domains are presented separately. Follow-ups were completed at T0, T4, and FU1 (one month after completion of 6 laser treatments). Scores at T0 are presented in the left bar (orange), at T4 in the middle bar (blue), and at FU1 on the right (pink). (**B**) Total score of FSFI. Scores at T0 are presented in the left bar (orange), at T4 in the middle bar (blue), and at FU1 on the right (pink).

Of 19 patients who initially received laser treatment, 18 opted to receive three additional treatments after unblinding (Figure 1). Among those who initially received sham, 10 opted to receive laser treatments (of whom 9 completed six sessions and 1 withdrew), while 5 chose to terminate their participation. Cumulatively, 27 women completed six laser treatments. The results of follow-up after six CO_2_ laser treatments for these 27 women are detailed in Figure 5, Figure 6 and Figure 7 and Tables S7 and S8.

#### 3.3.1. Primary Outcomes (Figure 5 and Figure 6, Tables S5–S8)

Dyspareunia significantly improved over time, with reduction evident at T6 (−2.44 ± 0.88, *p* = 0.005) and at FU1 (−2.24 ± 0.85, *p* = 0.008).

Vaginal dryness during sexual intercourse also showed a significant reduction at T4 (−1.93 ± 0.78, *p* = 0.013), T5 (−2.14 ± 0.78, *p* = 0.006), T6 (−3.30 ± 0.81, *p* < 0.001), and FU1 (−3.73 ± 0.84, *p* < 0.001).

Nevertheless, both primary outcome measures remained high after six laser sessions (Figure 5).

#### 3.3.2. Secondary Outcomes

Itch significantly decreased by T5 (−1.46 ± 0.51, *p* = 0.004), T6 (−1.15 ± 0.56, *p* = 0.039), and FU1 (−1.38 ± 0.54, *p* = 0.010).

Discomfort significantly improved at T5 (−1.81 ± 0.64, *p* = 0.005), T6 (−2.05 ± 0.58, *p* < 0.001), and FU1 (−2.06 ± 0.59, *p* < 0.001).

Burning significantly decreased at T5 (−1.14 ± 0.55, *p* = 0.037), T6 (−1.10 ± 0.52, *p* = 0.036), and FU1 (−1.32 ± 0.53, p = 0.013).

Daily dryness significantly decreased at T5 (−2.10 ± 0.76, *p* = 0.006), T6 (−2.34 ± 0.82, *p* = 0.004), and FU1 (−2.70 ± 0.76, p = 0.0004).

No significant improvement in dysuria and discharge were observed throughout the follow-up period.

Average VAS score: Significant reduction was seen by T5 (−1.31 ± 0.42, *p* = 0.002), T6 (−1.60 ± 0.45, *p* =0.004), and FU1 (−1.70 ± 0.46, *p* = 0.002), indicating an improvement in overall symptoms severity.

pH (Figure 6) values decreased significantly by FU1 (−0.54 ± 0.23, *p* = 0.017).

VHI (Figure 6): Significant improvements were observed at T3 (2.64 ± 0.96, *p* = 0.006), T4 (3.03 ± 0.92, *p* = 0.001), T5 (4.25 ± 0.93, *p* < 0.001), T6 (4.81 ± 0.86, *p* < 0.001), and FU1 (5.72 ± 0.96, *p* < 0.001), demonstrating a steady and substantial improvement over treatments.

Vaginal hydration (Figure 6) significantly improved at T4 (4.46 ± 1.46, *p* = 0.002) and FU1 (7.31 ± 1.87, *p* < 0.001.FSFI (Female Sexual Function Index, Figure 7)

In the FSFI, some domains showed improvement; however, this was not clinically significant:

Desire: an improvement was observed at T4 (0.68 ± 0.32, *p* = 0.037) but not in FU1.

Lubrication: a borderline improvement was observed at FU1 (0.97 ± 0.50, *p* = 0.054).

Satisfaction: an increase was observed at FU1 (1.02 ± 0.49, *p* = 0.038).

No significant improvements were found regarding arousal, orgasm, and pain domains throughout the follow-up period. In the total FSFI score, a borderline improvement was seen at T4 (4.24 ± 2.20, *p* = 0.054) and FU1 (4.33 ± 2.45, *p* = 0.078), but these did not reach statistical or clinical significance.

Adverse events of minor spotting and vaginal discomfort were reported in five patients in the treatment group and seven patients in the sham group (26.3% vs. 46.7%, *p* = 0.288). Two patients (one in each group) reported post-treatment cystitis necessitating antibiotics.

## 4. Discussion

The results of the first phase, which compared laser and sham treatments, targeting the most distressing symptoms reported by patients, dyspareunia, and intercourse dryness, showed no difference between laser and sham one month after the completion of three treatment sessions. Compared to baseline, changes were evident in both groups (defined as “time effect”), implying that sham therapy also elicits a response. These findings are consistent with a recently published RCT [34], which examined the efficacy of laser treatment versus sham in BC survivors combined with lubricants and vibratory stimulation over five consecutive sessions.

The GEE model revealed that laser treatment significantly reduced patient-reported daily dryness. This improvement was supported by objective measures, including increased vaginal hydration, as measured by the Schirmer test, elevated VHI-scores, and decreased pH (although the pH remained above 4.5, indicating a clinically insignificant change). Contrarily, laser treatment worsened itch, dysuria, burning, and discharge when compared to sham.

The results of the second phase, aimed at evaluating the effect of additional laser treatments, indicated that adding three more laser sessions seemed to improve outcomes, with a major limitation being the absence of a sham comparator, and the potential bias caused by patients’ expectations after initially experiencing sham procedure. However, even six sessions were not enough to promote the clinically required effect of eliminating GSM symptoms, especially dyspareunia and dryness during sexual intercourse.

GSM symptoms pose a significant concern in BC survivors [8]; first, most patients are menopausal at the time of diagnosis, whereas premenopausal women may experience early ovarian insufficiency due to chemotherapy or surgical removal [9,35,36]. Moreover, the growing use of antiestrogenic adjuvant therapy intensifies menopausal symptoms [8], especially with AI use [7].

Vaginal laser devices have been suggested as an alternative treatment for GSM, resulting in intensive marketing. Nevertheless, evidence regarding the efficacy of these treatments remains insufficient. Several RCTs have evaluated the efficacy of fractional CO_2_ lasers in postmenopausal women without BC, yielding conflicting results [25,26,27,28,29]. These trials included over 300 participants in total and utilized a similar treatment protocol involving three consecutive sessions. However, the primary and secondary outcomes varied among the trials, and the follow-up periods ranged from 4 weeks to 12 months. One trial found significant improvement in VHI and VAS at 12-week follow-up [25], while another trial [27] reported a significant improvement in GSM symptoms and FSFI at one month after treatment completion compared to the sham group. Another trial found no significant difference in symptom severity between the CO_2_ laser and sham groups after 12 months [28]. In a recent study by Page et al. [29], treatment results 12 weeks post laser or sham application were found to be comparable.

Non-randomized studies assessing vaginal laser therapy in BC survivors have suggested that the treatment is effective in addressing GSM symptoms, vaginal atrophy, and sexual function [14,15,16,17,18,19,20,21,22,23,24,37]. In a retrospective case series [17], BC survivors undergoing CO_2_ laser therapy reported significant symptom improvement, regardless of age or adjuvant therapy. Other studies have shown that the improvement in subjective and objective parameters can last up to 12–24 months after treatment [16,24,37]. No adverse effects were reported in these observational studies. Moreover, CO_2_ laser treatments in BC patients have been found to induce changes in vaginal inflammatory and modulatory cytokine patterns [38]. A recent RCT by Mension et al. [34], which included the usage of nonhormonal moisturizers and vaginal vibrator stimulation in all participants, compared 5 monthly sessions of either fractional CO_2_ laser or sham treatments. It found no significant differences between the groups at 1-month follow-up, with patients in both groups reporting improvement in FSFI and other subjective and objective outcomes.

The limited number of randomized studies and the heterogeneity among them hinder the ability to draw clinically significant conclusions regarding the actual benefit of laser treatments for GSM. Variations in follow-up periods and endpoints further contribute to the lack of consensus. Currently, there are no universally accepted and validated measures to evaluate the effectiveness of laser treatments for GSM, and the clinical relevance of the measured endpoints remains unclear.

In the current study, three CO_2_ laser treatments did not result in an improvement in intercourse dryness and dyspareunia compared to sham. However, it resulted in an improvement of daily dryness, supported by an objective measure, the Schirmer test, showing an actual effect on hydration. It is possible that higher energy levels than those utilized in this study may be required to achieve optimal therapeutic effects associated with clinical improvement. However, increasing the energy levels was not possible due to patients’ intolerance. Patients receiving continuous anti-estrogenic treatment may suffer from vaginal hypersensitivity due to severe epithelial thinning or from somatosensory aberrations produced by these treatments [39,40].

Further research is required to assess vaginal wall changes under antiestrogenic therapy, especially when testing energy-based treatment for GSM. Future RCTs should assess the number of laser sessions and the optimal laser energy levels required to achieve satisfactory clinical improvement in this population. This will enable the clarification of the long-term benefits and safety of this procedure.

The strengths of this study include the blinding of participants and the use of a full sham protocol to quantify the placebo effect. Additionally, the follow-up evaluation was conducted one month after treatment completion, avoiding the possible attenuation of the treatment’s impact over time. The participants represent a clinically realistic target population for intravaginal laser treatments. Lastly, we employed multiple outcome measures (both subjective and objective), including several assessment tools utilized in previous studies (i.e., VHI, pH, and FSFI), allowing for effective comparison across studies. Unlike Mension et al. [34], our study specifically focused on the effect of laser treatment without additional interventions, such as lubricants or vibrator stimulation.

Our study has limitations, mainly the small sample size and suboptimal laser energy levels used. Additionally, there was an unequal distribution of patients in the two groups due to the randomization method and the dropout of two patients from the sham group. Another limitation is the potential bias in the evaluation of patients, as the same physician who performed the treatment also assessed the VHI score, which is a subjective measure. However, this did not impact the co-primary outcome measures, including symptom scoring, pH measurement, and the Schirmer test. Achieving complete blinding in laser studies is challenging due to the associated pain, heat, odor, spotting, and discharge associated with the procedure. Although we implemented strategies to maximize blinding, we did not assess patients’ perception of their treatment assignment to confirm effective blinding.

## 5. Conclusions

The efficacy of energy-based treatment for GSM remains unestablished. Further evaluation of optimal treatment protocols, patient selection criteria, and long-term outcomes is needed before integrating fractional CO_2_ laser therapy into routine care for breast cancer survivors with GSM.

## Figures and Tables

**Table 1:** Patients’ characteristics.

Characteristics	Laser (n = 19)	Sham (n = 15)	*p* Value
Age	45 [40.75–53]	47 [42–54.5]	0.509
Tobacco use	1 (5.3%)	1 (5.6%)	>0.99
Comorbidities	4 (21.1%)	4 (26.7%)	>0.99
**Menopause cause**			0.493
Spontaneous	3 (15.8%)	5 (33.3%)	
Surgical	2 (10.5%)	3 (20.0%)	
Chemotherapy	9 (47.4%)	4 (26.7%)	
Medical	5 (26.3%)	3 (20.0%)	
**Years of menopause**			0.732
<2	6 (31.6%)	3 (20.0%)	
2–5	7 (36.8%)	6 (40.0%)	
>5	6 (31.6%)	6 (40.0%)	
**Gravity**	4 [2–4]	4 [3–4.5]	0.606
**Parity**	3 [2–3]	3 [2–3]	0.686
**Vaginal deliveries**	2 [1–3]	2 [0–3]	0.696
**Body mass index**	24.12 [22.3–25.3]	24.4 [22–28.0]	0.5374

## Data Availability

The data presented in this study are available on request from the corresponding author, including deidentified patient data, physical exam findings, questionnaires (VAS and FSFI), and study protocol. The data are available upon request from the corresponding author and will be shared with other researchers for metanalysis or review.

## Supplementary Materials

The following supporting information can be downloaded at: https://www.mdpi.com/article/10.3390/cancers17071241/s1, Figure S1: VAS scores at baseline; Table S1: The vaginal health index (VHI), Table S2: Treatment given for breast cancer, Table S3: Prior or current GSM treatment, Table S4: Parameters in phase 1 (comparative study, Laser vs Sham), Table S5: Results of the Female Sexual Function Index (FSFI) in Phase 1 (comparative study, Laser vs Sham), Table S6: The General Estimating Equation model (GEE) results- Phase 1 (comparative study) , Table S7: Comparison of outcome parameters at initial assessment (T0) and after six Laser treatments (FU1), n = 27. Delta for each parameter (presented as mean and standard deviation (SD)) after 6 laser treatments is calculated and presented as as $Delta={Value}_{T0}-{Value}_{EOP1}$, Table S8: The General Estimating Equation model (GEE) results- Six laser treatments

## Abbreviations

The following abbreviations are used in this manuscript:

AI	Aromatase inhibitors
BC	Breast cancer
EOP1	End of phase 1
FSFI	Female Sexual Function Index
GEE	Generalized estimating equations
GSM	Genitourinary syndrome of menopause
VAS	Visual Analogue Scale
VHI	Vaginal Health Index
