# Supplementary material for: CO2 Laser Therapy for Genitourinary Syndrome of Menopause in Women with Breast Cancer: A Randomized, Sham-Controlled Trial
Source: Cancers (Basel). 2025 Apr 6;17(7):1241. doi: 10.3390/cancers17071241 (PMC11988059; doi:10.3390/cancers17071241)
Supplement: Supplementary file 1 [file cancers-17-01241-s001.zip › cancers-3479227-supplementary.pdf]

Supplementary Materials

Figure S1

VAS scores at baseline

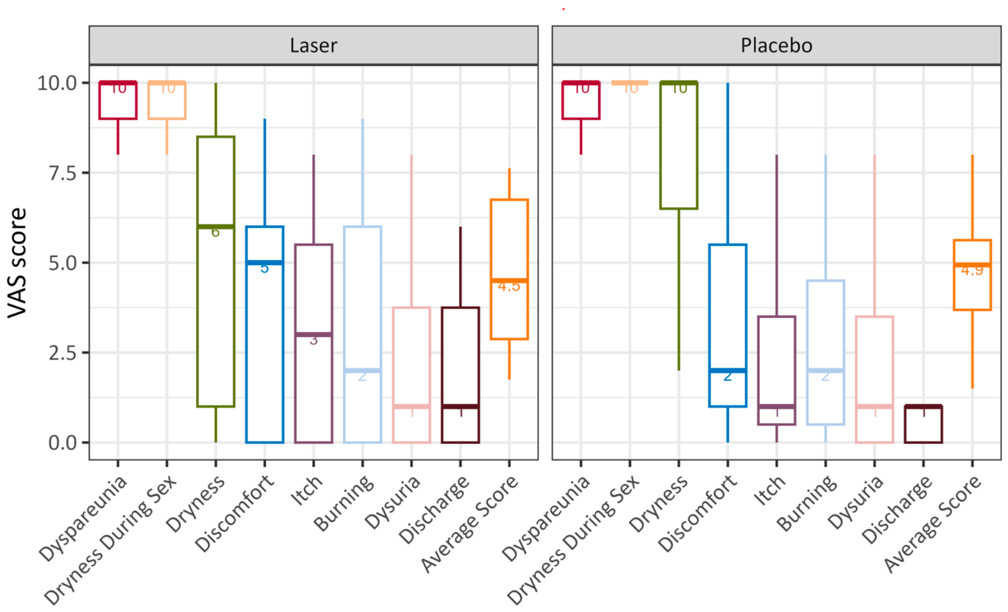

Table S1

The vaginal health index (VHI) (adapted from Bachmann et al [30]).

**Score**	**1**	**2**	**3**	**4**	**5**
**Elasticity**	None	Poor	Fair	Good	Excellent
**Fluid Volume (Pooling of Secretions)**	None	Scant amount, vault not entirely covered	Superficial amount, vault entirely covered	Moderate amount of dryness (small areas of dryness on cotton-tip applicator)	Normal amount (fully saturates on cotton-tip applicator)
**pH**	6.1 or above	5.6–6.0	5.1–5.5	4.7–5.0	4.6 or below
**Epithelial Integrity**	Petechiae noted before contact	Bleeds with light contact	Bleeds with scraping	Not friable, thin epithelium	Normal
**Moisture (Coating)**	None, surface inflamed	None, surface not inflamed	Minimal	Moderate	Normal

Table S2

Treatment given for breast cancer

BC Treatment Characteristics	Laser (n=19)	Sham (n=15)	p value
Previous BSO	6 (33.3%)	4 (26.7%)	0.722
**Adjuvant Therapy**			
Radiotherapy	16 (84.2%)	14 (93.3%)	0.613
Chemotherapy	16 (84.2%)	10 (66.7%)	0.417
Hormonal (anti estrogen)	16 (84.2%)	11 (73.3%)	0.672
Herceptin	2 (10.5%)	1 (6.7%)	>0.99
Biologic treatment	4 (21.1%)	3 (20.0%)	>0.99
**Hormonal treatment**			
Aromatase inhibitors (AI)	11 (57.9%)	10 (66.7%)	0.601
Tamoxifen	3 (15.8%)	3 (20.0%)	>0.99
GnRH agonists	9 (47.4%)	6 (40%)	0.667
Combined therapy	8 (42.1%)	8 (53.3%)	0.522
Current AI Treatment	15 (78.9%)	9 (60.0%)	0.276
AI treatment duration (months)	12 [6-18]	21 [12-36]	0.050

AI- Aromatase inhibitors, BC-breast cancer, BSO-bilateral salpingo-oophorectomy, GnRH-gonadotropin releasing hormone, GSM-Genitourinary Syndrome of Menopause

Table S3

Prior or current GSM treatment

Prior GSM treatment	Laser (n=19)	Sham (n=15)	p value
**Hormonal treatment prior to BC diagnosis**			
**Local**	2 (10.5%)	2 (13.3%)	
**Systemic**	2 (10.5%)	1 (6.7%)	>0.99
**Local and systemic**	1 (5.3%)	0 (0%)	
**None**	14 (73.7%)	12 (80.0%)	
**Washout period (months)**	18 [15-21]	24 [18-24]	0.637
**Current GSM treatment**			
**Daily use of vaginal lubricants**	2 (10.5%)	0 (0%)	0.492
**Current use of lubricants during intercourse**	13 (72.2%)	12 (80.0%)	0.699

BC-breast cancer, GSM-Genitourinary Syndrome of Menopause

Table S4- Parameters in phase 1 (comparative study, Laser vs Sham)

The parameters at baseline (T0) and after three CO2-laser or Sham sessions (End of Phase 1, EOP1) are presented as the mean and standard deviation (Sd). The difference in each parameter after Laser/Sham is calculated and presented as $Delta = Value_{T0} - Value_{EOP1}$.

Measure	Time	Laser (n=19)	Sham (n=15)	Total	p value
Dyspareunia (VAS)	T0	8.88 (1.82)	9.00 (1.55)	8.93 (1.69)	0.9357
	EOP1	7.25 (3.15)	6.82 (3.16)	7.07 (3.10)	0.7430
	Delta	1.62 (2.06)	2.18 (2.52)	1.85 (2.23)	0.6999
Intercourse Dryness (VAS)	T0	9.31 (1.35)	9.75 (0.45)	9.50 (1.07)	0.5392
	EOP1	7.06 (3.45)	7.08 (3.15)	7.07 (3.27)	0.9245
	Delta	2.25 (2.96)	2.67 (3.03)	2.43 (2.94)	0.6880
Dryness (VAS)	T0	5.26 (4.03)	7.67 (3.44)	6.32 (3.91)	0.0440
	EOP1	4.16 (3.50)	4.53 (3.72)	4.32 (3.55)	0.7533
	Delta	1.11 (4.34)	3.13 (4.03)	2.00 (4.27)	0.1337
Discomfort (VAS)	T0	3.74 (3.33)	3.53 (3.50)	3.65 (3.36)	0.9580
	EOP1	2.00 (2.11)	2.53 (2.88)	2.24 (2.45)	0.8166
	Delta	1.74 (2.38)	1.00 (3.57)	1.41 (2.93)	0.5279
Itch (VAS)	T0	3.26 (2.94)	2.70 (2.96)	3.01 (2.92)	0.6723
	EOP1	2.00 (2.52)	1.27 (1.49)	1.68 (2.13)	0.5357
	Delta	1.26 (2.18)	1.43 (2.57)	1.34 (2.32)	0.8175
Burning (VAS)	T0	2.89 (3.23)	2.67 (2.61)	2.79 (2.92)	0.8964
	EOP1	1.78 (1.99)	1.07 (1.58)	1.45 (1.82)	0.2550
	Delta	1.11 (2.52)	1.60 (2.38)	1.33 (2.43)	0.3315
Dysuria (VAS)	T0	2.44 (3.24)	2.40 (3.20)	2.42 (3.17)	0.9091
	EOP1	1.83 (2.36)	0.60 (1.35)	1.27 (2.04)	0.0515
	Delta	0.61 (2.23)	1.80 (3.26)	1.15 (2.76)	0.2044
Discharge (VAS)	T0	1.72 (2.05)	1.13 (1.81)	1.45 (1.94)	0.5927
	EOP1	2.22 (2.21)	1.53 (2.42)	1.91 (2.30)	0.2045
	Delta	-0.50 (2.26)	-0.40 (2.56)	-0.45 (2.36)	0.6231
Average VAS Score	T0	4.67 (2.06)	4.59 (2.02)	4.64 (2.01)	0.9585
	EOP1	3.38 (1.75)	2.97 (1.89)	3.20 (1.80)	0.4987
	Delta	1.30 (1.67)	1.62 (2.10)	1.44 (1.85)	0.7417
pH	T0	6.61 (0.92)	6.78 (0.72)	6.67 (0.84)	0.7282
	EOP1	6.16 (1.13)	6.44 (0.79)	6.27 (1.00)	0.7428
	Delta	0.45 (0.83)	0.34 (0.69)	0.40 (0.77)	0.5509
Schirmer test (millimeters)	T0	4.11 (4.01)	2.43 (3.50)	3.38 (3.83)	0.2154
	EOP1	10.11 (5.88)	5.43 (4.93)	8.06 (5.89)	0.0257
	Delta	-6.00 (6.03)	-3.00 (5.33)	-4.69 (5.84)	0.1175
VHI	T0	12.89 (2.78)	11.73 (2.12)	12.36 (2.53)	0.2876
	EOP1	16.67 (3.88)	14.60 (4.60)	15.73 (4.28)	0.0668
	Delta	-3.78 (3.47)	-2.87 (3.31)	-3.36 (3.38)	0.3823

T0- baseline assessment; EOP1- end of phase 1- assessment after 3 laser or Sham sessions; VAS-visual analog scale, VHI-vaginal health index. Significant p values are bold.

Table S5

Results of the Female Sexual Function Index (FSFI) in Phase 1 (comparative study, Laser vs Sham)

The parameters at baseline (T0) and after three CO2-laser or Sham sessions (EOP1) are presented as the mean and standard deviation (Sd). The difference in each parameter after Laser/Sham is calculated and presented as $Delta = Value_{T0} - Value_{EOP1}$.

Scale	Time	Laser (n=19)	Sham (n=15)	Total	p value
Arousal	T0	2.07 (1.50)	2.38 (1.56)	2.17 (1.50)	0.8154
	EOP1	2.83 (1.76)	2.47 (2.19)	2.71 (1.88)	0.7765
	Delta	-0.77 (1.43)	-0.08 (1.19)	-0.54 (1.37)	0.1699
Desire	T0	2.07 (1.07)	2.10 (0.87)	2.08 (0.99)	0.6863
	EOP1	2.87 (1.19)	2.17 (1.44)	2.63 (1.30)	0.2699
	Delta	-0.80 (0.82)	-0.07 (1.39)	-0.56 (1.08)	0.0937
Lubrication	T0	1.57 (1.41)	1.98 (1.57)	1.71 (1.45)	0.5649
	EOP1	2.25 (1.85)	2.15 (1.76)	2.22 (1.79)	0.9172
	Delta	-0.68 (1.44)	-0.17 (1.45)	-0.51 (1.43)	0.3657
Orgasm	T0	1.96 (1.65)	2.53 (1.85)	2.15 (1.71)	0.3787
	EOP1	3.16 (2.26)	2.40 (2.46)	2.90 (2.31)	0.3366
	Delta	-1.20 (2.34)	0.13 (1.50)	-0.76 (2.16)	0.1155
Pain	T0	1.44 (1.61)	1.51 (0.79)	1.47 (1.38)	0.3969
	EOP1	1.69 (1.79)	1.24 (1.43)	1.54 (1.67)	0.5778
	Delta	-0.24 (1.10)	0.27 (1.28)	-0.07 (1.16)	0.2789
Satisfaction	T0	2.27 (1.56)	3.07 (1.48)	2.53 (1.55)	0.2239
	EOP1	2.82 (2.04)	2.76 (1.55)	2.80 (1.86)	0.9794
	Delta	-0.56 (1.86)	0.31 (2.02)	-0.27 (1.92)	0.5699
FSFI Summary	T0	11.37 (7.52)	13.58 (7.29)	12.10 (7.38)	0.5715
	EOP1	15.62 (8.59)	13.18 (9.48)	14.81 (8.79)	0.6434
	Delta	-4.25 (7.00)	0.39 (7.32)	-2.70 (7.31)	0.1649

T0- baseline assessment; EOP1- end of phase 1- assessment after 3 laser or Sham sessions. Significant p values are bold.

Table S6

The General Estimating Equation model (GEE) results- Phase 1 (comparative study)

The model explores the influence of treatment and time on the various outcomes, accounting for repeated measurements per subject, and focusing on treatment effect and time effects as compared to baseline. It adjusts for repeated measurements within each patient (SE-standard error)

Scale	Measure	Time	Estimate	SE	p value	Interpretation
**VAS**	**Dyspareunia**	TimeT1	-0.305	0.486	0.5310	NS
		TimeT2	-1.037	0.585	0.0766	borderline
		TimeT3	-1.561	0.641	0.0149	Improved
		TimeEOP1	-1.891	0.639	0.0031	Improved
		Laser	0.123	0.432	0.7767	NS
	**Intercourse dryness**	TimeT1	-0.139	0.299	0.6421	NS
		TimeT2	-1.659	0.526	0.0016	Improved
		TimeT3	-2.336	0.572	0.0000	Improved
		TimeEOP1	-2.379	0.616	0.0001	Improved
		Laser	-0.028	0.396	0.9442	NS
	**Dryness**	TimeT1	0.102	0.936	0.9131	NS
		TimeT2	-0.134	0.829	0.8717	NS
		TimeT3	-1.303	0.879	0.1381	NS
		TimeEOP1	-2.000	0.877	0.0225	Improved
		Laser	-1.303	0.548	0.0175	Sig improvement
	**Itch**	TimeT1	-0.124	0.724	0.8642	NS
		TimeT2	-0.825	0.637	0.1948	NS
		TimeT3	-0.677	0.677	0.3170	NS
		TimeEOP1	-1.338	0.606	0.0272	Improved
		Laser	0.840	0.393	0.0326	Opposite effect
	**Dysuria**	TimeT1	0.208	0.881	0.8132	NS
		TimeT2	-0.735	0.731	0.3145	NS
		TimeT3	-0.602	0.735	0.4128	NS
		TimeEOP1	-1.147	0.640	0.0733	borderline
		Laser	1.259	0.422	0.0029	Opposite effect
	**Discomfort**	TimeT1	-0.428	0.835	0.6084	NS
		TimeT2	-0.507	0.771	0.5114	NS
		TimeT3	-0.910	0.771	0.2377	NS
		TimeEOP1	-1.412	0.702	0.0445	improved
		Laser	0.087	0.483	0.8569	NS
	**Discharge**	TimeT1	0.371	0.594	0.5328	NS
		TimeT2	0.305	0.569	0.5923	NS
		TimeT3	0.482	0.489	0.3243	NS
		TimeEOP1	0.455	0.513	0.3754	NS
		Laser	1.140	0.362	0.0016	Opposite effect
	**Burning**	TimeT1	-0.583	0.717	0.4161	NS
		TimeT2	-0.678	0.663	0.3065	NS
		TimeT3	-0.627	0.668	0.3477	NS
		TimeEOP1	-1.301	0.587	0.0268	Improved
		Laser	0.964	0.367	0.0086	Opposite effect
	**Average Score**	TimeT1	0.308	0.524	0.5568	NS

		TimeT2	-0.639	0.469	0.1732	NS
		TimeT3	-1.076	0.488	0.0274	Improved
		TimeEOP1	-1.441	0.455	0.0015	improved
		Laser	0.419	0.302	0.1651	NS
**pH**		TimeT1	0.226	0.228	0.3221	NS
		TimeT2	-0.082	0.201	0.6816	NS
		TimeT3	-0.209	0.213	0.3272	NS
		TimeEOP1	-0.455	0.223	0.0411	improved
		Laser	-0.315	0.137	0.0214	Sig improvement
**VHI**		TimeT1	0.947	0.607	0.1185	NS
		TimeT2	1.529	0.729	0.0360	Improved
		TimeT3	2.706	0.752	0.0003	Improved
		TimeEOP1	3.346	0.826	0.0001	Improved
		Laser	2.257	0.500	0.0000	Sig improvement
**Schirmer test**		TimeEOP1	4.735	1.146	0.0000	improved
		Laser	3.236	1.125	0.0040	Sig improvement
**FSFI**	**Arousal**	TimeEOP1	0.539	0.454	0.2348	NS
		Laser	0.025	0.502	0.9603	NS
	**Desire**	TimeEOP1	0.556	0.305	0.0689	borderline
		Laser	0.333	0.328	0.3090	NS
	**Lubrication**	TimeEOP1	0.511	0.434	0.2393	NS
		Laser	-0.158	0.459	0.7301	NS
	**Orgasm**	TimeEOP1	0.756	0.542	0.1634	NS
		Laser	0.089	0.591	0.8804	NS
	**Pain**	TimeEOP1	0.074	0.408	0.8558	NS
		Laser	0.189	0.380	0.6192	NS
	**Satisfaction**	TimeEOP1	0.267	0.456	0.5584	NS
		Laser	-0.367	0.454	0.4189	NS
	**FSFI Summary**	TimeEOP1	2.702	2.168	0.2127	NS
		Laser	0.111	2.322	0.9618	NS

Table S7
Comparison of outcome parameters at initial assessment (T0) and after six Laser treatments (FU1), n=27

Delta for each parameter (presented as mean and standard deviation (SD)) after 6 laser treatments is calculated and presented as $Delta = Value_{T0} - Value_{EOP1}$

	T0	FU1	Delta	p Value
**Dyspareunia (VAS)**	8.14 (2.55)	5.90 (3.24)	2.45 (2.72)	0.876
**Intercourse Dryness (VAS)**	8.45 (2.41)	4.72 (3.35)	3.73 (3.63)	0.122
**Dryness (VAS)**	4.94 (3.85)	2.24 (2.12)	3.13 (3.66)	0.815
**Discomfort (VAS)**	3.21 (3.15)	1.15 (1.38)	2.11 (2.98)	0.639
**Itch (VAS)**	2.38 (2.58)	1.00 (1.64)	1.59 (2.55)	0.979
**Burning (VAS)**	2.06 (2.74)	0.74 (1.29)	1.46 (2.69)	0.426
**Dysuria (VAS)**	1.61 (2.69)	1.72 (2.79)	-0.33 (2.79)	**0.022**
**Discharge (VAS)**	2.73 (3.31)	1.75 (1.97)	0.10 (2.20)	0.303
**Average VAS Score**	3.92 (2.13)	2.22 (1.48)	1.97 (1.95)	0.129
**pH**	6.54 (0.86)	6.00 (0.90)	0.52 (1.02)	0.627
**Schirmer test (mm)**	4.69 (4.41)	12.00 (8.84)	-7.44 (8.99)	0.302
**VHI**	13.68 (3.70)	19.40 (3.35)	-6.45 (3.50)	0.135

T0- baseline assessment; FU1-follow-up 1, (after 6 laser treatments); VAS-visual analog scale; mm-millimeter VHI-vaginal health index.
Significant p values are bolded.

Table S8

The General Estimating Equation model (GEE) results- Six laser treatments

The model examines the cumulative treatment effect on various outcomes over six treatments and during the initial follow-up, relative to baseline. It adjusts for repeated measurements within each patient.

Scale	Measure	term	Estimate	Std.error	p.value	Interpretation
	Dyspareunia	TimeT1	0.14	0.64	0.8256	NS
	Dyspareunia	TimeT2	-0.31	0.73	0.6679	NS
	Dyspareunia	TimeT3	-0.49	0.70	0.4862	NS
	Dyspareunia	TimeT4	-1.18	0.74	0.1102	NS
	Dyspareunia	TimeT5	-1.37	0.80	0.0864	Borderline
	Dyspareunia	TimeT6	-2.44	0.88	0.0054	Improved
	Dyspareunia	TimeFU1	-2.24	0.85	0.0081	Improved
	Intercourse dryness	TimeT1	0.12	0.61	0.8450	NS
	Intercourse dryness	TimeT2	-0.32	0.65	0.6233	NS
	Intercourse dryness	TimeT3	-0.86	0.69	0.2126	NS
	Intercourse dryness	TimeT4	-1.93	0.78	0.0131	Improved
	Intercourse dryness	TimeT5	-2.14	0.78	0.0064	Improved
	Intercourse dryness	TimeT6	-3.30	0.81	0.0000	Improved
	Intercourse dryness	TimeFU1	-3.73	0.84	0.0000	Improved
	Daily dryness	TimeT1	0.28	0.95	0.7652	NS
	Daily dryness	TimeT2	0.69	0.92	0.4521	NS
	Daily dryness	TimeT3	-0.03	0.88	0.9696	NS
	Daily dryness	TimeT4	-0.61	0.89	0.4952	NS
	Daily dryness	TimeT5	-2.10	0.76	0.0058	Improved
	Daily dryness	TimeT6	-2.34	0.82	0.0043	Improved
	Daily dryness	TimeFU1	-2.70	0.76	0.0004	Improved
	Itch	TimeT1	-0.06	0.61	0.9224	NS
	Itch	TimeT2	0.01	0.65	0.9872	NS
	Itch	TimeT3	0.31	0.70	0.6523	NS
	Itch	TimeT4	-0.85	0.60	0.1597	NS
	Itch	TimeT5	-1.46	0.51	0.0039	Improved
	Itch	TimeT6	-1.15	0.56	0.0394	Improved
	Itch	TimeFU1	-1.38	0.54	0.0098	Improved
	Dysuria	TimeT1	0.39	0.82	0.6303	NS
	Dysuria	TimeT2	0.11	0.71	0.8781	NS
	Dysuria	TimeT3	0.47	0.72	0.5182	NS
	Dysuria	TimeT4	-0.28	0.60	0.6333	NS
	Dysuria	TimeT5	-0.63	0.58	0.2782	NS
	Dysuria	TimeT6	-0.35	0.61	0.5736	NS
	Dysuria	TimeFU1	0.11	0.72	0.8735	NS
	Discomfort	TimeT1	-0.01	0.76	0.9870	NS
	Discomfort	TimeT2	-0.31	0.76	0.6818	NS
	Discomfort	TimeT3	-0.71	0.72	0.3285	NS
	Discomfort	TimeT4	-1.26	0.66	0.0577	Borderline
	Discomfort	TimeT5	-1.81	0.64	0.0050	Improved

	Discomfort	TimeT6	-2.05	0.58	0.0004	Improved
	Discomfort	TimeFU1	-2.06	0.59	0.0005	Improved
	Discharge	TimeT1	1.10	0.91	0.2260	NS
	Discharge	TimeT2	0.81	0.73	0.2684	NS
	Discharge	TimeT3	0.83	0.59	0.1588	NS
	Discharge	TimeT4	0.56	0.54	0.3036	NS
	Discharge	TimeT5	1.18	0.69	0.0885	Borderline
	Discharge	TimeT6	1.11	0.66	0.0895	Borderline
	Discharge	TimeFU1	0.11	0.53	0.8311	NS
	Burning	TimeT1	0.04	0.67	0.9533	NS
	Burning	TimeT2	0.15	0.68	0.8219	NS
	Burning	TimeT3	0.15	0.67	0.8179	NS
	Burning	TimeT4	-0.70	0.57	0.2211	NS
	Burning	TimeT5	-1.14	0.55	0.0367	Improved
	Burning	TimeT6	-1.10	0.52	0.0361	Improved
	Burning	TimeFU1	-1.32	0.53	0.0125	Improved
	Average Score	TimeT1	0.41	0.52	0.4387	NS
	Average Score	TimeT2	0.06	0.51	0.9093	NS
	Average Score	TimeT3	-0.18	0.50	0.7201	NS
	Average Score	TimeT4	-0.76	0.46	0.1007	NS
	Average Score	TimeT5	-1.31	0.42	0.0018	Improved
	Average Score	TimeT6	-1.60	0.45	0.0004	Improved
	Average Score	TimeFU1	-1.70	0.46	0.0002	Improved
pH		TimeT1	0.25	0.23	0.2765	NS
		TimeT2	-0.01	0.22	0.9647	NS
		TimeT3	-0.11	0.23	0.6306	NS
		TimeT4	-0.24	0.25	0.3346	NS
		TimeT5	-0.21	0.23	0.3627	NS
		TimeT6	-0.40	0.24	0.0969	Borderline
		TimeFU1	-0.54	0.23	0.0169	Improved
VHI		TimeT1	0.86	0.89	0.3336	NS
		TimeT2	1.22	0.92	0.1839	NS
		TimeT3	2.64	0.96	0.0057	Improved
		TimeT4	3.03	0.92	0.0010	Improved
		TimeT5	4.25	0.93	0.0000	Improved
		TimeT6	4.81	0.86	0.0000	Improved
		TimeFU1	5.72	0.96	0.0000	Improved
Schirmer test		TimeT4	4.46	1.46	0.0022	Improved
		TimeFU1	7.31	1.87	0.0001	Improved
FSFI	Arousal	TimeT4	0.57	0.46	0.2147	NS
	Arousal	TimeFU1	0.70	0.50	0.1585	NS
	Desire	TimeT4	0.68	0.32	0.0365	Improved
	Desire	TimeFU1	0.33	0.33	0.3126	NS
	Lubrication	TimeT4	0.71	0.45	0.1126	NS
	Lubrication	TimeFU1	0.97	0.50	0.0539	Borderline
	Orgasm	TimeT4	1.03	0.55	0.0590	Borderline

	Orgasm	TimeFU1	0.75	0.55	0.1760	NS
	Pain	TimeT4	0.56	0.44	0.1987	NS
	Pain	TimeFU1	0.56	0.46	0.2235	NS
	Satisfaction	TimeT4	0.68	0.46	0.1422	NS
	Satisfaction	TimeFU1	1.02	0.49	0.0380	Improved
	FSFI Sum	TimeT4	4.24	2.20	0.0539	Borderline
	FSFI Sum	TimeFU1	4.33	2.45	0.0776	Borderline
